# First Contact Practitioners’ (FCPs) and General Practitioners’ Perceptions Towards FCPs Delivering Vocational Advice to Patients with Musculoskeletal Conditions: A Qualitative Investigation of the Implementation Potential of the I-SWAP Initiative

**DOI:** 10.1007/s10926-021-09992-5

**Published:** 2021-07-09

**Authors:** Benjamin Saunders, Nadine E. Foster, Jonathan C. Hill, Gail Sowden, Nicola Evans, Annette Bishop, Siobhan Stynes, Krysia Dziedzic, Laura Campbell, Gabrielle Rankin, Paula Salmon, Gwenllian Wynne-Jones

**Affiliations:** 1 Primary Care Centre of Excellence Versus Arthritis, School of Medicine, Keele University, Staffordshire, UK; 2 STARS Research and Education Alliance, Surgical Treatment and Rehabilitation Service, The University of Queensland and Metro North Hospital and Health Service, Herston, QLD Australia; 3 Connect Health, Newcastle upon Tyne, UK; 4 Midlands Partnership Foundation NHS Trust, Haywood Hospital Spinal Interface Service, Staffordshire, UK; 5 Chartered Society of Physiotherapy (CSP), London, UK; 6 Mid Cheshire Hospital Foundation Trust, Leighton Hospital, Crewe, UK

**Keywords:** Musculoskeletal pain, Primary health care, Physical therapists, Work, Implementation science

## Abstract

*Purpose* Musculoskeletal (MSK) pain is a common cause of work absence. The recent SWAP (Study of Work And Pain) randomised controlled trial (RCT) found that a brief vocational advice service for primary care patients with MSK pain led to fewer days’ work absence and provided good return-on-investment. The I-SWAP (Implementation of the Study of Work And Pain) initiative aimed to deliver an implementation test-bed of the SWAP vocational advice intervention with First Contact Practitioners (FCP). This entailed adapting the SWAP vocational advice training to fit the FCP role. This qualitative investigation explored the implementation potential of FCPs delivering vocational advice for patients with MSK pain. *Methods* Semi-structured interviews and focus groups were conducted with 10 FCPs and 5 GPs. Data were analysed thematically and findings explored using Normalisation Process Theory (NPT). *Results* I-SWAP achieved a degree of ‘coherence’ (i.e. made sense), with both FCPs and GPs feeling FCPs were well-placed to discuss work issues with these patients. However, for many of the FCPs, addressing or modifying psychosocial and occupational barriers to return-to-work was not considered feasible within FCP consultations, and improving physical function was prioritised. Concerns were also raised that employers would not act on FCPs’ recommendations regarding return-to-work. *Conclusion* FCPs appear well-placed to discuss work issues with MSK patients, and signpost/refer to other services; however, because they often only see patients once they are less suited to deliver other aspects of vocational advice. Future research is needed to explore how best to provide vocational advice in primary care settings.

## Background

Musculoskeletal (MSK) pain is a common cause of work absence, and early intervention is encouraged to prevent negative health and economic consequences that result from longer-term absence and work loss [1, 2]. However, accessing vocational advice and support is challenging for many people. For example, in the UK, it is estimated that only a third of employees have access to occupational health services [2], which leaves the majority looking towards primary care health professionals for occupational support. There are challenges for healthcare professionals in meeting this need, with training and education in managing health and work being paramount [3].

In order to explore new models of care to improve access to vocational advice and support, the SWAP (Study of Work And Pain) randomised controlled trial (RCT) tested the effectiveness of a brief vocational advice (VA) service in general practice, providing support for patients struggling or absent from work due to MSK pain. This VA service was provided by physical therapists trained to identify obstacles to working with MSK pain and to support patients to overcome these obstacles [4]. Results showed that the VA intervention was effective, leading to an average reduction in work absence of five days per employed patient over four months with a Return on Investment (ROI) of £49 per £1 invested [5]. Following the success of the SWAP trial, methods to implement the findings were considered, guided by the knowledge that the VA service was best provided to patients early in their work absence, and patients found VA from physical therapists acceptable. Furthermore, physical therapists have demonstrated a willingness and ability to address work issues with patients with MSK pain [6].

In the UK, a new model of care involves First Contact Practitioners (FCPs) being utilised in primary care as the first point-of-contact for MSK presentations, in order to (i) ensure the provision of early, specialist assessment; (ii) save time and resources within primary care teams; (iii) encourage diversification in the primary care workforce; and (iv) improve collaborative interdisciplinary working [7]. The FCP role is typically carried out by advanced practice physical therapists working within general practices. FCPs are able to assess, diagnose, manage and discharge patients without the need for a GP consultation. Within this model of care there is an expectation of the management of work issues relating to MSK health and the consideration of the need for any workplace adaptations to meet the individual’s needs [8]. It was felt that this group could be well-placed to deliver brief VA as they will see patients early on, have experience in managing MSK pain, and on average offer consultations of 20–30 min compared to 10-min for GP consultations [9].

The I-SWAP (Implementation of the Study of Work And Pain) initiative aimed to deliver an implementation test-bed of the VA intervention used in the SWAP RCT, with employed patients with MSK pain consulting a FCP. This entailed adapting the VA training for physical therapists previously used in the SWAP trial [4], for use by FCPs. Details of the adapted VA training for FCPs in I-SWAP is provided below. The I-SWAP initiative was carried out in conjunction with the National Health Service (NHS) England phase 3 FCP national evaluation [10], which investigated patient reported experience and outcomes in 40 FCP services across England.

This paper reports on a qualitative investigation of the implementation potential of FCPs delivering VA to patients with MSK pain. This involved collaboration between academics and participating FCPs, as well as GPs, to evaluate the key barriers and facilitators of FCPs delivering VA in consultations.

### VA Training Provided to FCPs in I-SWAP

The I-SWAP initiative was conducted in two FCP services in the Midlands and North West of England. Seventeen FCPs were trained to deliver VA across 38 general practices, over 9 months. The training delivered in the SWAP trial [4] was adapted through two workshops with FCPs, which focused on defining the profile of patients accessing the FCP service, and examining the content of the SWAP training to identify topics to take into the I-SWAP initiative and those which were either redundant (i.e. the FCPs already had an understanding/training in these areas) or were too complex for FCPs to fit within their consultations [Table 1].

**Table 1:** Content of the VA training in I-SWAP

*Value of work*
Is work good for your health?
Portraying positive message about work
*The relationship between health and work*
*Common myths and changing beliefs*
What works when providing vocational advice
*Obstacles to returning work*
*What do we say to patients?*
Practical recommendations
Use of allied health professions (AHP) health and work report
*Mental illness*
*Sickness policies and benefits*
*AHP health and work report*
*Managing unhelpful beliefs*
Patient information
Reassurance

A tool available to FCPs in I-SWAP that had not been available during the SWAP trial was the Allied Health Professional (AHP) Health and Work Report: (http://www.ahpf.org.uk/files/AHP%20Health%20and%20Work%20report.pdf). The AHP Health and Work Report enables AHPs to provide recommendations to a patient’s GP and employer which may be used to help keep them in work if possible, or be signed off from work for a period of time if necessary. The use of this as a structured method of exploring VA with patients and communicating with GPs and employers was developed.

FCPs were provided with a two-hour, face-to-face training session on the provision of VA to MSK patients (delivered by GWJ); this compares to a 3-day programme with 4th day refresher session delivered in SWAP. The training was interactive, with FCPs asked to contribute examples of delivering VA and applying knowledge gained during training to reflect on how their current practice could be enhanced. Practical training was also provided in completing the AHP Health and Work Report to develop confidence in using this document as a framework for delivering VA as part of a routine consultation.

## Methods

The qualitative investigation included focus groups and one-to-one semi-structured interviews, conducted in September–October 2019, with FCPs participating in I-SWAP, and GPs working in the same practice as participating FCPs (*n* = 10 FCPs, 5 GPs). GPs were included to investigate the implications of adding VA to FCP consultations for primary care more broadly, including any impact on GPs’ discussions with patients about work issues.

A participatory approach was taken whereby clinicians were treated as collaborators rather than participants, working with the academic team to jointly explore the implementation potential of the I-SWAP initiative. Involving stakeholders in the evaluation of healthcare services has been argued to be ‘an important catalyst for improvement’ [11]. As such, one of the authors (PS) is an FCP who helped adapt the training from the SWAP trial to fit the FCP role, and also delivered VA to patients in the I-SWAP initiative. PS also took part in an interview, thus allowing the perspectives of an FCP implementing the initiative to have an influence on the presentation of the findings.

Given that this was an investigation of the implementation potential of I-SWAP, conducted in collaboration with participating FCP services, and not a research study, NHS research ethical approval was not required, but agreement of service managers was sought to interview staff. Usual ethical principles and procedures were followed, including gaining informed consent from clinicians who took part in focus groups/interviews, as detailed below.

### Theoretical Framework

Incorporating VA into consultations may involve FCPs working in different ways. Changing healthcare practice raises diverse challenges, and examining such challenges using a theoretically-underpinned approach can extend the scope of purely descriptive approaches [12]. As such, we drew on Normalisation Process Theory (NPT) [13], which provides a framework both for planning and understanding the implementation of healthcare initiatives, in particular, why some are accepted and more successfully embedded in routine practice than others [13]. NPT comprises four components, which provide a lens through which to interpret our findings: coherence (or sense-making); cognitive participation (or engagement); collective action (work done to enable the intervention to happen); and reflexive monitoring (formal and informal appraisal of the benefits and costs of the intervention).

### Data Collection

FCPs and GPs were invited to take part via email, which included an information sheet. Upon reply a suitable time/place for the focus group or interview was arranged. Focus groups/interviews were led by BS, an experienced qualitative researcher, and were conducted at the clinicians’ workplaces (aside from one FCP interview via telephone), and lasted between 23 and 40 min.

Whilst the FCPs were all participating in I-SWAP, the GPs were less familiar with the initiative. A short animation was therefore shown to GPs prior to the focus group discussion explaining the aims of I-SWAP and content of the VA training provided to FCPs. Focus groups/interviews were informed by topic guides [see supplementary file]; topics included how successfully or otherwise VA provision was seen to ‘fit’ within the FCP role, and discussion of barriers and enablers to inter-disciplinary communication about patients’ occupational issues. Topic guides were used flexibly, allowing for any unexpected findings to be further explored. All clinicians provided written informed consent before the start of focus groups/interviews, except the telephone interview where consent was audio-recorded.

### Data Analysis

Audio-recordings of focus groups/interviews were transcribed and anonymised. A two-stage analysis framework was used; first, thematic analysis, and second, mapping the identified themes onto the four NPT components. Anonymised transcripts were first coded on a line-by-line basis by BS, using the qualitative software program Nvivo 12, to identify concepts inductively. Analysis drew on the constant comparison method [14], looking for connections within and across focus groups/interviews, and across codes, highlighting data consistencies and variation. Analysis began with the FCP data and then mapped the views of GPs against those of the FCPs, to allow for direct comparison between the two. Data analysis was discussed at regular meetings between team members from different disciplinary backgrounds (BS: social science; GWJ: occupational health research and nursing; JH and GS: complex intervention development and testing, and physical therapy; NE: implementation science), ensuring inter-disciplinary perspectives on the data. Input was also provided by members of the I-SWAP steering group, which included two FCPs and a ‘patient champion’, who was involved throughout the development of the initiative. This led to the identification of three main themes. Themes were then explored in relation to how well they ‘fitted’ with the four NPT components [15]. ‘Fit’ was indicated by how closely the findings within each theme correlated with the components and parameters of NPT, and therefore how usefully the findings could be interpreted through the lens of these components. If findings do not appear to ‘fit’ well with a theoretical framework, this would be akin to trying to fit a square peg into a round hole, as the findings would not be adequately explained by the theory. This is one reason that it may be advantageous to analyse qualitative data inductively to begin with, before then exploring the data in relation to theory, as this can allow for the identification of findings that may not correlate with the components of that theory. This was not the case with our data, however, and a strong level of ‘fit’ was observed between our themes and the NPT components − as outlined later in the Discussion − meaning none of the findings fell outside of the parameters of NPT, and the identified themes could be usefully explained through the lens of this theory. Once analysis was completed, a summary of the findings was presented to six members of the I-SWAP Patient and Public Involvement and Engagement (PPIE) group, who provided views on the implications of the findings for future services. In what follows we outline the characteristics of the clinicians who took part, before reporting the key themes.

## Clinician Characteristics

Fifteen clinicians took part across two focus groups and two one-to-one interviews─ five GPs from the same GP practice, and 10 FCPs from the two FCP services taking part in I-SWAP. Clinicians had a range of clinical experience; GPs ranged from newly qualified to over 20 years in clinical practice; FCPs ranged from 6 years qualified as a physical therapist to over 20 years in clinical practice. In addition to their FCP role, all participating FCPs also worked in community or secondary care physical therapy roles. Six FCPs were female, and four male; and one GP was female, and four male.

## Principle Findings

The three main themes identified were as follows:

1. Feasibility of incorporating VA within the current FCP role
2. Perceptions towards the use of the AHP health and work report
3. Implications of the provision of VA by FCPs for inter-disciplinary working

### Theme 1: Feasibility of Incorporating VA Within the Current FCP Role

FCPs perceived discussing occupational issues with patients as being part of their FCP role, and reported already having these conversations with patients to varying extents prior to I-SWAP:

*You’re always discussing how [patients] can be as involved in their activities as they can be with their problem. So you’re always discussing work to some extent…the vast majority of patients are keen to stay in paid work, mainly for financial reasons…even without I-SWAP. (FCP interview 2)*

GPs felt that FCPs were well-placed to discuss work with patients, both because FCPs have longer consultations, and GPs recognised that FCPs may possess more specialist MSK knowledge than themselves:

*I think (the FCP working in the practice) is very well-placed (to provide VA) because he has more time, longer consultations…plus he’s probably got a bit more knowledge on musculoskeletal things than GPs. So for those two reasons, he’s better placed. (GP participant 1, Focus group 2)*

However, whilst they reported discussing work issues, most FCPs felt that their capacity to address barriers to RTW in any great depth was limited due to the time constraints of FCP consultations. As a result, they prioritised trying to improve patients’ physical function, and where more complex work issues were identified, patients were signposted or referred to other services for these issues to be addressed, e.g. community physical therapy or their GP. This was particularly the case in relation to the management of psychosocial barriers to RTW, such as anxiety and distress about RTW. The lack of opportunity to build rapport with patients in a single consultation was also identified by FCPs as a barrier to addressing RTW barriers:

*FCP 5: I think it would be hard within the timeframe of 30 min to do any sort of greater depth…that might be something more if you refer on to be looked at*

*FCP 3: Definitely the psychosocial element. You can’t in half an hour*

*FCP 8: Yeah, you haven’t really had chance to build up any sort of proper rapport with the patient to gain their trust to access that more psychosocial side (Focus group 1)*

For these reasons, most FCPs did not feel they were fully able to implement the VA training they had received into their practice. They suggested that the community NHS physical therapy role would be more suited to addressing and modifying barriers to RTW, given that patients are often offered a number of consultations:

*We might only see them as a one off…different to in my other physiotherapy role because you see them more regularly, you can see the progress they're making…you can make a more informed decision about how long they may or may not be off work, whereas a one off assessment, it can be quite difficult. (FCP 6, Focus group 1)*

Another factor in dealing with psychosocial barriers to RTW was that several FCPs felt their clinical training was primarily in managing what they termed ‘low level’ psychosocial obstacles, such as patients expressing pain-related fear of movement. They distinguished these types of psychosocial issues from more significant distress which may be causing or contributing to work absence. Where such distress resulted in patients experiencing anxiety or depression, they saw GPs as being better suited to address this:

*We see psychosocial [factors] pretty much with every patient to a certain degree, but it’s more low level things like fear avoidance…most people after injury are fearful of movement...But if someone’s clearly off work for an extended period and there's a significant psychological issue, I don’t think we’re properly trained to be able to say, for instance, ‘I think you need CBT [cognitive behavioural therapy] and it’ll be in the form of this’. (FCP 9, Focus group 1)*

GPs also saw themselves as better suited to manage patients for whom more significant psychological distress was driving work absence, and this was framed as part of a joint approach between themselves and the FCPs:

*I think that’s it’s a joint approach…[the FCP] could give advice about what can and can’t be done with certain musculoskeletal things, what to expect, and we deal with the psychological aspect of it. I mean he can deal with the psychological aspect as well but I think if it’s a bit too complicated for him to deal with it, then it’s a joint approach really. (GP 5,Focus group 2)*

There were some divergent data, however, as two particularly experienced FCPs highlighted that because they have longer consultations than GPs, they felt well-placed to identify and address mental health issues related to the patient’s pain at an early stage, potentially preventing these issues from escalating into more severe mental health problems:

*If there is a mental health issue…I think we’re really well-placed to deal with that and potentially well-placed to stop that from progressing into a bigger mental health episode. (FCP interview 2)*

### Theme 2: Perceptions Towards the Use of the AHP Health and Work Report

All FCPs highlighted limitations of the AHP health and work report (from here on AHP report), particularly that it is not compulsory for employers to adhere to and therefore lacks legitimacy. This was seen as a significant barrier to its use:

*It’s not a legally binding document and the employer doesn’t have to do anything at all because it is purely advice; whereas, the GP’s one carries clout. Ours carries absolutely nothing. If it actually made somebody do something, I think we’d be more tempted to do it. If they actually really need time off work, you’d fill that in and then you’d still be going to the GP to say, ‘Can you sign them off?. (FCP interview 1)*

GPs echoed concerns about the lack of legitimacy of the AHP report, expressing doubts as to whether employers would abide by the recommendations:

*If we've done it, the employer has to try and accommodate it I think, whereas if it is your friendly physio…they don’t have to abide by it because it’s voluntary…if you’ve got a good employer they will do, but if you’ve got a care assistant who’s on five quid an hour or whatever it is they’re on, the margins are so tight, they can’t decide to give them an extra friend to help do lifting, and if it’s not legally [binding], they won’t*. *(GP 3, Focus group 2)*

The length of the AHP report and the time it would take to complete was also seen as a barrier to its use, and as a result, only one FCP reported having used it:

*It’s too long and too much to fill in. In the format that we’ve got now, it’s just too time consuming and too much detail*

*Interviewer: What might encourage you to make more use of it?*

*Possibly more tick boxes, and a little bit of free text. There are very definite things that we discuss; if you could tick it and then just put a very minimal statement next to it, that might be more user-friendly than just a blank sheet that you have to fill lots in. (FCP interview 1)*

GPs also felt its use could result in a heavier workload for them, because when administering a fit note, they would also have to incorporate the FCP’s recommendations:

*It may actually make more work for us because instead of doing a fit note that says, ‘back pain, off for three weeks’, we have to do ‘fit for work with limited duties, back pain, provided he does the following, blah, blah, blah’ and actually that’s a paragraph of typing that we don’t have to do if we sign them off sick. I mean it would be nice to have [the AHP report] there so you can just copy out what someone else has said but you will still have to do it, unless the FCP can issue the fit note. (GP 4, Focus group 2)*

### Theme 3: Implications of Adding VA to the FCP Role for Inter-Disciplinary Working

Both clinician groups reported already having regular communication about specific patients prior to I-SWAP, but said these did not commonly include discussions about work issues:

*I talk to [the in-practice FCP] about patients. I can’t say that I've had a conversation with him about work issues. It’s usually complicated cases, that he thinks there's something else going on, not just a musculoskeletal issue, there's a more systemic disease. (GP 5, Focus group 2)*

Given this regular communication, neither group could foresee the addition of VA to the FCP role significantly impacting their communication about patients. GPs also reported that they did not feel there would be a change in their own workload, as they felt that some patients would be less willing to engage in discussions about RTW planning with FCPs due to a desire to be signed off from work. They anticipated that these patients would still consult with the GP to obtain a fit note; therefore, GP MSK workload would not be reduced:

*GP3: His advice is very good for people who want to go back to work…but there are people who don’t want to go back to work because of various issues like psychological issues, pain issues, complicated issues.*

*GP5; Yes, because some of them will book an appointment for that fit note. So some of them, having seen [the FCP], will come back to see us to tell us. (Focus group 2)*

In contrast, however, one FCP who had worked within the same general practice for four years, reported that her role in addressing work issues had resulted in fewer fit notes being given by GPs in that practice:

*Our GPs will categorically say that they sign far fewer sick notes now than they ever did. I’ve worked with those guys for four years now and we definitely have changed discussions around work very much in that particular practice. (FCP interview 1)*

FCPs reported not usually making contact with employers or occupational health departments to discuss individual patients, and were not confident that I-SWAP would change this. This was partly due to the reported variability in the nature of different workplaces and the support they offer to employees. In the case of some employers, it was not felt that they would be willing to communicate with the FCP or to engage with their recommendations:

*You have to have the employer wanting to work with you. A lot of the industries, say, a supermarket warehouse, it’s all timed. You only get X amount of pay if you do X amount. I don’t think they would want to talk to me in the slightest…other employers are very, very flexible. If we recommend X, Y and Z, they tend to be really supportive...But by actually ringing them up, I don’t think I’d get very far. (FCP interview 1)*

## Discussion

The findings presented indicate that within this new model of primary care delivery, whilst both FCPs and GPs felt that FCPs were well-placed to identify and discuss work issues with patients, there are barriers to delivering other aspects of VA, such as addressing or modifying psychosocial barriers to RTW, related to the practicalities and perceived scope of the FCP role. To more fully understand the identified barriers and facilitators, we considered the findings through the lens of the four core components of Normalisation Process Theory (NPT).

### Coherence, Cognitive Participation, Collective Action and Reflexive Monitoring

A degree of coherence of the I-SWAP initiative was observed in that, for both clinician groups, discussing work issues with patients ‘made sense’ within the FCP role. However, the work conversations FCPs reported already having with patients were seen to be ‘distinguishable’ [16] from the VA training FCPs received as part of I-SWAP [Table 1, earlier] and they felt that they lacked the time and opportunity to really address obstacles to RTW with patients; therefore, in this sense the initiative lacked coherence with existing ways of working.

A barrier to FCPs’ cognitive engagement and participation in delivering VA was that some felt they needed to prioritise efforts to improve the patient’s physical function over specifically addressing work-related problems (though it was acknowledged that improving physical function was part of helping patients to RTW). As a result, they felt unable to undertake the ‘collective action’ required fully engage with the I-SWAP initiative. However, the FCPs who felt their longer consultation times put them in a better position to identify and address psychosocial RTW barriers than GPs, generally expressed greater motivation for engaging in providing VA. FCPs who had not felt able to implement the brief VA in the I-SWAP initiative as a result found it difficult to reflect on or appraise its use. All FCPs and GPs did see potential future benefits of the addition of VA to MSK care for patients, but FCPs in particular felt that community or secondary care physical therapy consultations would be more suitable to provide VA than FCP consultations.

#### Comparison with Previous Literature

The finding in this investigation that FCPs routinely discuss work issues with patients with MSK pain shows similarity with those from the NHS England phase 3 FCP national evaluation [10], in which interviews with FCPs showed that they would consider work-related issues with every patient of working age. However, of the 89 patients in the evaluation that reported MSK-related days-off-work, under half (45%) reported receiving advice about work, suggesting that, similar to our findings, VA was not being fully delivered. This is despite qualitative research into patients’ preferences having shown that patients do want to discuss their ability to continue working with MSK pain, and the timing of RTW, with their FCP [17].

Our finding that some FCPs felt less confident in managing cases where significant distress may be causing or contributing to work absence is reflected elsewhere in the literature. Whilst not focusing specifically on managing work absence, a recent qualitative interview study with 10 FCPs reported a similar finding that FCPs felt that they had not had the required training to manage mental health issues raised by patients [18].

Previous research has identified GP concern regarding the legitimacy of AHPs in providing recommendations for workplace amendments or sickness absence [6]. Similarly, qualitative research carried out as part of the SWAP RCT found GPs to be sceptical about whether employers would adhere to recommendations from physical therapists providing VA [19]. However, the difference in our findings is that the legitimacy of FCPs in fulfilling this role was not in question, as GPs felt FCPs were well-placed to deliver VA. Instead, it was specifically the legitimacy of the AHP health and work report (which was not included as part of the VA training in SWAP) that was questioned.

Clinicians in our investigation also felt that the likelihood of employers adopting the FCPs’ RTW recommendations were dependent on the nature of different workplaces, including what occupational support, if any, they offered their employees. This variability in occupational and RTW support is reflected in the broader occupational rehabilitation literature, and in relation to management of MSK pain specifically. Whilst the I-SWAP initiative was UK-based, RTW support has been found to be lacking in certain occupation types, such as manual and service roles, even in countries that have a generally higher level of occupational health provision when compared to the UK [20]. For instance, a Danish study found that 20 people with back pain in manual job roles (e.g. maintenance of parks, recreational areas, cemeteries, road service) reported a lack of workplace support, and any workplace adjustments that were considered to help them stay in work or RTW were felt by participants to be driven by economic considerations rather than employees’ needs [21]. This correlates with the perspectives of some of the clinicians in our investigation towards the UK context; they expressed the view that workplace adaptations are less likely to be adopted by UK employers for patients in lower-paid or manual job roles, due to cost considerations.

Employers’ communication with healthcare professionals about employee work absence has been argued to be an important factor in helping to facilitate RTW for people with MSK pain [20]. However, FCPs in our study reported not usually making contact with employers, as they felt that employers would not want to speak with them, even in the case of those employers who were generally more supportive and willing to adopt their recommendations. Research in other allied health professions has made similar finding; a qualitative survey of 143 Occupational Therapists (OTs) found that the OTs did not commonly have contact with employers about their employees’ work absence, and when they did it was likely to have been initiated by the OT [22]. Given the finding elsewhere that workplace support can play a significant positive role in influencing people’s confidence to RTW after absence due to MSK pain [23], we would argue that identifying ways to encourage employers to engage with RTW support and communicate with healthcare professionals, particularly in relation to individuals in manual and service job roles, may be an important area to target for future interventions.

#### Implications for Practice

As outlined earlier (see Background), in the previous successful SWAP trial, physiotherapists were trained to provide a new VA service within general practice [5], whereas the current I-SWAP initiative involved training FCPs to deliver VA as part of their existing FCP role. As reflected in the findings, the fact that FCPs were asked to deliver VA alongside their other roles and responsibilities − rather than focusing specifically on VA provision as in the SWAP trial − could help to explain why the delivery of VA appeared less successful in the I-SWAP initiative. FCPs reported a lack of time in a one-off consultation, alongside other consultation goals, to fully address or modify obstacles to RTW with patients, or to build therapeutic rapport, which they felt was necessary for exploring psychosocial barriers to RTW. It may be that community or secondary care physical therapy services are more suitable settings for addressing and modifying biopsychosocial barriers to RTW. All of the FCPs who participated in the evaluation also worked in these other settings, therefore it is not the case that they saw themselves as being the wrong professional group to deliver VA to patients, but rather that they could more successfully deliver VA in these other settings, given the ability to offer more consultations and develop a greater therapeutic rapport with patients to facilitate more detailed identification and modification of RTW obstacles.

In addition to time constraints, some FCPs reported that despite the training received in I-SWAP, they did not feel well-equipped to address some psychosocial barriers to RTW, such as work-related distress and anxiety. However, those who were more experienced in the FCP role felt more comfortable managing these issues. It may be that delivery of this component of VA is dependent on the experience-level of the clinician, and that further training in managing work-related anxiety and distress is needed for less experienced FCPs to feel confident to fully deliver VA. In the UK NHS, the recently developed ‘Roadmap to Practice’ for FCPs and Advanced Practitioners [8] may help to address this, through supporting FCPs to meet the key capabilities related to the FCP role, which include advising patients on strategies and adaptations to help them stay in work or RTW.

Finally, barriers that were identified to the use of the AHP health and work report, in terms of its legitimacy and time to complete it, could be addressed through changes to the structure and role of the report, to encourage greater engagement with it. These changes could include enabling FCPs to use this report to sign patients off from work, as part of the FCP competencies progressing to advanced practice, and subsequently reduce MSK workload for GPs. However, it is important to note that not all FCPs in this investigation reported feeling comfortable with the idea of doing this, and therefore further training may be required.

#### Strengths and Limitations

A strength is the parallel investigation of views of the two different clinician groups, which included FCPs from two different services. The multi-disciplinary team involved in data analysis was also a strength, which increases the trustworthiness of the findings presented. Additionally, the use of NPT enabled us to develop a more robust understanding of the implementation potential of adding brief VA to the FCP role.

A limitation is that we were not able to gain patients’ or employers’ views of the I-SWAP initiative, which would have provided additional insight, but was not possible within the scope of the investigation.

## Conclusion

The findings presented suggest that FCP consultations may not be the right setting to fully deliver VA. Whilst it appears that FCPs are well-placed to deliver some aspects of VA to patients with MSK pain; namely, identification of barriers to RTW, screening for psychosocial obstacles to recovery and signposting/referring patients; other aspects such as addressing and modifying obstacles to RTW may be beyond the scope of the present FCP role.

Further work in this area could include continuing to collaborate with FCPs to further modify the VA training based on the views presented here, to find the most effective way to fit this within the FCP role. Alternatively, testing implementation of VA in community physical therapy would allow for the evaluation of whether elements such as addressing and modifying obstacles to RTW, are more suited to this setting.

## Data Availability

In line with the Standard Operating Procedures in place at Keele School of Medicine, where this study was conducted, data is archived at a dedicated location within the Keele network. A request to access archived data can be made by completion of a Data Transfer Request form, which can be accessed by contacting the school directly: School of Medicine, Keele University, Staffordshire, ST5 5BG; Tel: + 44 (0) 1782 733,905.
